# “I am the embodiment of an anorexic patient's worst fear”: Severe obesity and binge eating disorder on a restrictive eating disorder ward

**DOI:** 10.1111/cob.12398

**Published:** 2020-09-10

**Authors:** Kaitlyn N. Matthews, Maria Psihogios, Elizabeth Dettmer, Cathleen Steinegger, Alene Toulany

**Affiliations:** ^1^ Department of Paediatrics University of Toronto Toronto Ontario Canada; ^2^ Department of Paediatrics Michael Garron Hospital Toronto Ontario Canada; ^3^ Division of Adolescent Medicine Hospital for Sick Children Toronto Ontario Canada

**Keywords:** binge eating disorder, adolescence, health equity

## Abstract

Adolescents who have been diagnosed with an eating disorder commonly have comorbid mental health conditions which have a significant impact on illness trajectory and may even limit access to effective treatment. Current models of eating disorder care focus mainly on treatment for patients diagnosed with restrictive eating disorders with fewer options available for those with binge eating disorder. We describe a case of an adolescent living with severe, complex obesity and binge eating disorder, presenting in a mental health crisis, admitted to an in‐patient unit primarily for patients being treated for restrictive eating disorders such as anorexia nervosa. This case report describes multiple challenges that arose in admitting such a patient on a ward specializing in the treatment of restrictive eating disorders and highlights the need for equitable and more accessible care for patients living with all types of eating disorders.

## 1 INTRODUCTION

Psychiatric comorbidities are important predictors of impending hospitalization for adolescents diagnosed with eating disorders.^1^ Binge eating disorder (BED) is widely known to have severe consequences on physical health, but is also associated with significant adverse emotional outcomes.^2^ This report highlights voids in health care that surfaced when an adolescent female living with BED, severe obesity and psychiatric comorbidities was admitted to an in‐patient ward primarily focused on the treatment of restrictive eating disorders.

## 2 CASE DESCRIPTION

A 17‐year‐old female living with extreme BED and obesity presented to hospital with aggressive behaviour towards her primary caregiver (grandmother). Despite being followed in an interdisciplinary obesity management programme, her binge eating behaviours had resulted in a 45 kg weight gain over 12 months, resulting in numerous medical complications. Comorbid psychiatric diagnoses included anxiety disorders, post‐traumatic stress disorder and obsessive compulsive disorder. Prior to admission she resided in a group home with intensive psychological supports. Much of her therapeutic work at the group home focused on processing trauma and increasing effective coping skills using dialectical behaviour therapy and trauma informed approaches. However, due to limited resources, patients were sent home on weekends. Removed from the structure and extra support the group home provided, she reverted to ineffective strategies to gain emotional control such as binge eating and aggressively rejecting any boundaries set by her caregiver.

The patient was admitted to a ward designed for individuals diagnosed with restrictive eating disorders as no acute care treatment options existed for BED. The main goals of admission were symptom interruption, safety planning and care coordination. With structured meal times, interruption of bingeing was successfully achieved within 72 hours. Much of the admission (25 days) was then, however, dedicated to coordinating a discharge plan that would allow for intensive mental health support, while also ensuring safety for the patient's caregiver. Numerous barriers to finding a suitable treatment programme for an adolescent living with BED, complex obesity and mental health comorbidities surfaced. She could not return home with her grandmother due to safety concerns and, as a result, could also not return to the group home or attend an outpatient day programme. No residential programmes for youth living with BED existed. Ultimately, she was discharged to a residential home for youth living with mental health concerns that was not specific to eating disorders. The lack of long‐term care options for youth with BED and comorbid mental health concerns was striking.

Many learning points stemmed from the juxtaposition of admitting an adolescent living with severe obesity and BED to a restrictive eating disorder unit. Logistically, there were challenges related to medical equipment including unsuitable gown sizes, blood pressure cuffs and a scale incapable of recording higher weights. Applicability of usual ward protocols requiring all meals to be finished and supplementing calories for food refusal were questioned by unit staff.

The patient herself demonstrated insight into this stating, “I could see the nurses struggle to come up with a plan to control my eating habits as I was the polar opposite of all their past and present patients.” Additionally, the question of whether admitting a patient living with severe obesity would psychologically affect the patients admitted with restrictive eating behaviours, and vice versa, was also raised.

## 3 DISCUSSION

Many opportunities for reflection emerge from the unique context of admitting an adolescent living with severe obesity and BED to a restrictive eating disorders unit, and the subsequent barriers that surfaced in ascertaining an appropriate discharge plan for such a patient. First and foremost, this case draws attention to the significant and disheartening lack of suitable residential and in‐patient services for youth living with complex eating disorders. Further limiting treatment options, having both an eating and mental health disorder concomitantly is often considered an exclusion criterion for intensive mental health programmes. This case report especially raises the issue of lack of provision of such services for young people living with severe eating disorders that are not restrictive in nature, such as that of complex binge eating disorder and associated severe obesity. One might hypothesize that this is linked to the manner by which the differing eating disorder types are often perceived; while significant weight loss characteristic of restrictive eating disorders comes with acute medical risks, the complications of obesity are often perceived as more chronic in nature. Nevertheless, this case exemplifies how the sequelae of obesity have the potential to contribute to profound morbidity and psychiatric concerns associated with over‐eating disorders, that can in and of themselves, be quite acute and severe.^3^

The relative difference in in‐patient services for different types of eating disorders also potentially draws attention to the significant bias against obesity in medicine.^4^ There is evidence that in‐patient and residential treatment may be beneficial for symptom interruption and weight stabilization in individuals living with severe obesity and BED.^5^, ^6^, ^7^, ^8^, ^9^ One study aimed to compare the long‐term effects of the delivery of a residential cognitive‐behavioural treatment programme for weight loss in patients living with severe obesity, with and without BED.^8^ The residential programme, delivered out of Italy, consisted of 21 days of dietary, physical activity and cognitive behavioural therapy interventions. Among the many positive outcomes, the number of binge eating episodes in those participants with a diagnosis of BED decreased significantly.^8^ Another similar in‐patient rehabilitative programme, consisting of a multidisciplinary approach combining nutritional and physical rehabilitation with psychological and educational interventions, showed statistically significant reductions in body weight, body mass index and weight circumference for participants at discharge, compared to admission.^7^ In addition, the tendency to engage in binge eating behaviours was significantly reduced during the programme.^7^ While specialized in‐patient wards and residential programmes already exist internationally to serve the needs of individuals living with severe obesity and complex eating disorders such as BED,^5^ this is not the case currently in Canada.

This case secondly calls attention to the difficulties that arise in managing the needs of a patient living with severe obesity, binge eating disorder and complex mental health challenges, on an in‐patient unit that primarily cares for individuals diagnosed with restrictive eating disorders. In addition to the logistic challenges that surfaced with regards to medical equipment, as well as situations where the appropriateness of the restrictive eating disorder treatment protocols were called into question, concerns about admitting an adolescent living with obesity and BED to a unit where the majority of patients present as severely underweight, and the potential psychological impact this might have on the patients involved, also emerged. This concern was exemplified, for instance, when staff expressed that “it would not be helpful for [the patient's] emotional and psychological health to see such thin adolescents.” Research has explored the potential psychological dynamics that might come into play when individuals living with obesity engage with individuals who are underweight, and vice versa.^10^, ^11^, ^12^ For instance, research shows that when patients with anorexia or bulimia are shown photos of a variety of body types, they in fact tend to negatively evaluate individuals with obesity, rather than idealize ultra‐thin individuals.^10^, ^11^, ^12^ The patient herself spoke to the potential for psychological impact of being on a ward primarily for patients diagnosed with restrictive eating disorders when she shared, during a morning assessment, “I am the embodiment of an anorexic patient's worst fears. Is it really good for them to see me?”

**FIGURE 1:**
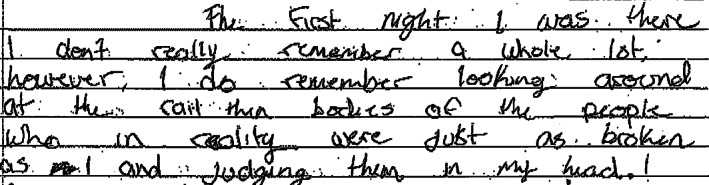
Excerpt from patient testimony written 3 months after the admission described herein

As healthcare practitioners, our view of eating disorders may sometimes be too narrowly focused on restrictive eating disorders. Although the differences between restrictive and binge eating disorders are highlighted herein, one cannot disregard the many similarities that also exist when one considers the inherent complexities in treating severe and complicated eating disorders. For instance, importance of an interdisciplinary approach, a focus on normalized eating, and the impact of psychosocial factors on prognosis are universal aspects of management. In addition, the underpinnings of both restrictive and binge eating disorders are presumed to be similar in that individuals living with either are more likely to have experienced trauma and use eating, or the lack of it, to cope and distance themselves from emotional pain. The profound distress shared by patients living with either type of eating disorder was captured when the patient herself reflected on her experience of the admission sharing, “I do remember looking around at the rail thin bodies of the people who, in reality, were just as broken as I" (Figure 1).

## 4 CONCLUSION

This case highlights barriers to accessing care for adolescents living with BED, but can be more widely applied to any adolescent living with a complex eating disorder with comorbid mental health challenges. It challenges the current presiding model of care serving adolescents with BED and complex obesity in Canada, which largely includes limited outpatient services, despite evidence for the benefits of in‐patient and residential programmes in meeting the needs of this population. Implementing a “mixed economy” approach to mental health services, consisting of not only robust outpatient services, but also established in‐patient and residential services, to support children and adolescents with complex mental health needs^13^ would seem to serve adolescents living with BED and complex obesity well. Importantly, this case also inspires reflection among healthcare providers when it comes to the way eating disorders are viewed in general; it encourages broadening such views to include others in addition to the restrictive type. Through the unique perspective of the expressed thoughts of the patient herself, the challenges that were inherent to this context were brought to light, as well as, the potentials to learn and improve to better meet the needs of adolescents with complex eating disorders and mental health challenges.

## CONFLICT OF INTEREST

No conflict of interest was declared.
